# Lag-Specific Transfer Entropy for Root Cause Diagnosis and Delay Estimation in Industrial Sensor Networks

**DOI:** 10.3390/s25133980

**Published:** 2025-06-26

**Authors:** Rui Chen, Shu Liang, Jian-Guo Wang, Yuan Yao, Jing-Ru Su, Li-Lan Liu

**Affiliations:** 1 College of Electronic and Information Engineering, Tongji University, Shanghai 200092, China; 2310887@tongji.edu.cn (R.C.); sliang@tongji.edu.cn (S.L.); 2 School of Mechatronical Engineering and Automation, Shanghai University, Shanghai 200072, China; yxy96@shu.edu.cn (J.-R.S.); lancy@shu.edu.cn (L.-L.L.); 3 Department of Chemical Engineering, National Tsing Hua University, Hsinchu 300044, Taiwan

**Keywords:** industrial sensors, lag-specific transfer entropy, root cause diagnosis, time delay, causality analysis

## Abstract

Industrial plants now stream thousands of temperature, pressure, flow rate, and composition measurements at minute-level intervals. These multi-sensor records often contain variable transport or residence time delays that hinder accurate disturbance analysis. This study applies lag-specific transfer entropy (LSTE) to historical sensor logs to identify the instrument that first deviates from normal operation and the time required for that deviation to appear at downstream points. A self-prediction optimization step removes each sensor’s own information storage, after which LSTE is computed at candidate lags and tested against time-shifted surrogates for statistical significance. The method is benchmarked on a nonlinear simulation, the Tennessee Eastman plant, a three-phase separator test rig, and a full-scale blast furnace line. Across all cases, LSTE locates the disturbance origin and reports propagation times that match known process physics, while significantly reducing false links compared to classical transfer entropy.

## 1. Introduction

The convergence of information technology and traditional manufacturing has turned modern plants into large-scale sensor networks in which thousands of temperature, pressure, flow rate, and composition transmitters report data at minute- or even second-level intervals [1,2]. Because these instruments are distributed across units, pipelines, and utility systems, any local disturbance detected by one sensor can propagate through material, energy, or control flows and appear—often after a time delay—in the readings of many others. Such propagation, if not recognized early, may degrade product quality, raise operating costs, or even threaten personnel safety [3]. Root cause diagnosis (RCD) therefore focuses on locating the first sensor that records an abnormal deviation so that operators can determine whether the problem lies in the instrument itself or in the underlying process [4].

In classical control terminology, a disturbance is defined as “any variable that causes the controlled variable to deviate from set point” [5]. Regardless of whether a disturbance reflects a genuine fault or simply a routine load or feed change, the material, energy, and control links that connect the sensors often remain intact, so the disturbance still propagates through the network along those predefined causal paths. By analyzing those paths, we can trace abnormal signals back to their origin [6].

Early RCD tools—such as adjacency matrices [7] and signed directed graphs (SDGs) [8]—model these links with first-principle knowledge of every unit operation. Such mechanism-based approaches work well for small systems, but become difficult to build and maintain for today’s high-dimensional sensor networks. Data-driven methods avoid detailed process descriptions by extracting the structure directly from archived measurements [9].

Machine-learning versions of those data-driven methods have produced good results, yet they often demand large, well-labeled datasets and complex training procedures [10]. This has renewed interest in statistical causality measures—notably Granger causality (GC) [11,12] and transfer entropy (TE) [13,14]—that work with moderate data volumes and minimal prior information. Nevertheless, no single method is best for every plant, so the choice must match the sensor network and operating regime.

Among these alternatives, TE is valued for capturing nonlinear interactions [15,16]. As an information-theoretic measure [17], it evaluates the conditional mutual information between the future of one sensor and the delayed past of another, given the target sensor’s own history; a positive value implies a directional flow of information from the source sensor to the target sensor [18]. It is important to note that a high transfer entropy (TE) value between two variables does not directly imply a causal relationship. Rather, TE quantifies the information flow or directional influence from one variable to another. Additional statistical significance tests are necessary to determine whether a TE value is meaningful—only statistically significant TE values may be interpreted as indicative of potential causal influence, rather than definitive proof of causality. Researchers have proposed many TE extensions. Symbolic TE (STE) reduces noise sensitivity for non-stationary data [19]; Partial TE (PTE) distinguishes direct from indirect links [20]; and further variants combine the two ideas or examine multiple time scales [13,21]. These versions, however, often require large sample sizes [22], and may perform poorly when strong linear relations co-exist with nonlinear ones [23]. To reduce the cost of nonlinear RCD, a kernel-sample equivalence method using TE was proposed and can identify the root cause sensor with less computation [24]. Papana et al. [16] proposed direct causality measures based on variable selection and dimensionality reduction techniques to reduce computational complexity, and systematically evaluated the performance of several bivariate and multivariate causality measures in the time domain. A multi-block Bayesian network driven by Direct Transfer Entropy (DTE) was built to detect feedback loops and locate the source sensor [25]. More recently, Information Granulation DTE (IGDTE) has grouped data into coarse granules before computing DTE, cutting the runtime while preserving accuracy [26].

Moreover, time lags naturally arise as disturbances propagate through interconnected process units [27], and these delays are crucial for understanding the dynamic behavior of complex sensor networks [28]. Given that industrial process data often exhibit both linear and nonlinear dependencies, and that time delay information is essential for accurate fault diagnosis, we propose a root cause diagnosis approach based on lag-specific transfer entropy (LSTE), originally introduced in [27], and tailor it to industrial process scenarios involving complex time–delay interactions. In this study, the goal is to develop an algorithm capable of analyzing time-delayed causal relationships among the variables in industrial processes, with the aim of achieving accurate root cause identification. This objective is accomplished by quantifying the information gain between variable pairs under varying time lag conditions and applying statistical significance testing. Compared to conventional lag-agnostic methods, the use of LSTE enables a more precise and informative characterization of the dynamic interactions within complex process systems. Specifically, the algorithm first removes each sensor’s self-predictable component with past self-prediction optimization (SPO). It then scans candidate lags and selects the one that maximizes transfer entropy, delivering both the delay and the strength of every causal link. This joint view of lag and causality traces the disturbance path in detail and pinpoints the originating sensor. The feasibility of the proposed method is demonstrated through applications to numerical simulations, the Tennessee Eastman Process (TEP), and the Three-Phase Flow (TPF) process. Additionally, LSTE is used to conduct an in-depth analysis of the time lag causal relationship between the blast furnace molten iron temperature and the related operating variables.

## 2. Fundamental Concepts

### 2.1. Foundations of Information Theory

TE originates from the concept of information entropy, which was introduced by Shannon in 1948 [17] to quantify information content. The entropy of a random variable X is defined as follows:

(1) $$H(X)=\sum_{i=1}^{n}p(x_{i}){log}_{2}p(x_{i}).$$

where $x_{i}$ denotes the *i*th measurement value of the random variable *X* and $p(x_{i})$ denotes the probability observing $X=x_{i}$. In practice, systems are typically composed of multiple interrelated variables. These interdependencies form a network of interactions. To capture the uncertainty within such systems, the entropy concept can be extended to multivariate settings.

For example, the joint entropy of two random variables X and Y is as follows:

(2) $$H(X,Y)=-\sum_{i=1}^{n}\sum_{j=1}^{n}p(x_{i},y_{j}){log}_{2}p(x_{i},y_{j}),$$

where $p(x_{i},y_{j})$ denotes the joint probability of $X=x_{i}$ and $Y=y_{j}$. The conditional entropy H(Y|X) represents the residual uncertainty in Y given the knowledge of X, and is defined as follows:

(3) $$H(Y|X)=-\sum_{i=1}^{n}\sum_{j=1}^{n}p(x_{i},y_{j}){log}_{2}p(y_{j}|x_{i}),$$

where $p(y_{j}|x_{i})$ is the conditional probability of $Y=y_{j}$ when given $X=x_{i}$.

### 2.2. Transfer Entropy

Let $X=\left[x_{1},x_{2},\ldots ,x_{n}\right]$ and $Y=\left[y_{1},y_{2},\ldots ,y_{n}\right]$ be two time series. The transfer entropy from *X* to *Y*, denoted as ${TE}_{X\to Y}$, is given using the following:

(4) $${TE}_{X\to Y}=\sum p(y_{t+1},y_{t}{,x}_{t})\log\frac{p(y_{t+1}|{y_{t},x}_{t})}{p(y_{t+1}|y_{t})},$$

where $p(y_{t+1},y_{t}{,x}_{t})$ denotes the joint probability density function, p(·|·) denotes the conditional probability density function, and $y_{t+1}$ denotes the measurement of Y at a future moment in time. In addition,$x_{t}=\left[x_{t},x_{t-\tau },\ldots ,x_{t-(d-1)\tau }\right]$ and $y_{t}=[y_{t},{\;y}_{t-\tau },\;y_{t-2\tau },\ldots ,y_{t-(l-1)\tau }]$ are the state space reconstruction vectors of variables X and Y, respectively, where d and l are the embedded dimension of variables X and Y, respectively. If τ is the embedded delay, then the length of time series reconstruction is $n^{\prime }=n-max\left\{\left(d-1\right)\tau ,\left(l-1\right)\tau \right\}$. Since causality and interaction are defined as properties of the system rather than scalar time series, the corresponding state space of the interacting system must be reconstructed from scalar time series before causality detection can be carried out. Generally, we choose d=l or d=1. The calculation of TE can also be expressed in terms of entropy as follows:

(5) $${TE}_{X\to Y}=-H\left(y_{t+1}|y_{t},x_{t}\right)+H\left(y_{t+1}|y_{t}\right)=-H(y_{t+1}{,y}_{t},x_{t})+H\left(y_{t},x_{t}\right)+H\left(y_{t+1},y_{t}\right)-H\left(y_{t}\right)$$

It can be seen from Equation (5) that the calculation of TE indicates whether the information entropy of $y_{t+1}$ is affected by $x_{t}$, which is consistent with Wiener’s causality theory. That is, if variable Y could be predicted more accurately using the past value of variable X, then variable X affects variable Y.

In addition, the TE is directional. If ${TE}_{X\to Y}>0$, it means that the information is transferred from variable X to variable Y; if ${TE}_{X\to Y}<0$, the information is passed from variable Y to variable X; and if ${TE}_{X\to Y}=0$, there is no causal relationship between the two variables.

## 3. Methodology

### 3.1. Principles of LSTE

There is a natural delay in the transmission of information between variables. The traditional TE method cannot accurately describe the system’s information interaction by using only the amount of information transferred at a future time point. Therefore, the concept of time lag is introduced into TE [29], i.e.,

(6) $${TE}_{X\to Y}=\sum p(y_{t+h},{y_{t},x}_{t})\log\frac{p(y_{t+h}|y_{t},x_{t})}{p(y_{t+h}|y_{t})},$$

where h denotes the time delay over which the influence spreads from X to Y; the transfer entropy value at this delay quantifies the amount of information transferred from X to Y at lag h. By adjusting the value of h, TE can adapt to different time delays among variables, making it more aligned with real-world dynamics and allowing the maximum TE value to indicate the causal relationship between variables.

However, Equation (6) violates the best self-prediction requirement implied by Wiener’s principle, and thus the value of h obtained may not reflect the true time lag. Wiener’s principle requires that the past of X provides additional information about the future of Y, beyond what is already provided by the past of Y. If the latter is underestimated, the inferred information transfer from X to Y will be exaggerated. Therefore, the past of Y must be optimally estimated—this is referred to as self-prediction optimization (SPO). From an information-theoretic perspective, “self-prediction” corresponds to the information storage of Y. Underestimating this storage leads to the overestimation of the transfer entropy from X to Y.

Assuming that Y satisfies SPO at $h_{0}$, where $h_{0}$ is the optimal time delay for the self-prediction of $y_{t}$ using $y_{t-h_{0}}$. For X, the time index t is replaced by t−h, and Equation (6) can be rewritten as follows:

(7) $${TE}_{X\to Y}(h)=\sum p(y_{t},{y_{t-h_{0}},x}_{t-h})\log_{2}\frac{p(y_{t}|y_{t-h_{0}},x_{t-h})}{p(y_{t}|y_{t-h_{0}})}.$$

As shown in [29], if Equation (7) is used to express causality, then $h_{0}$ must be 1. This setting eliminates the information storage of Y’s past and avoids mistaking it for information transferred from X. Accordingly, the TE that satisfies SPO is as follows:

(8) $${SPOTE}_{X\to Y}\;(h)=\sum p(y_{t},y_{t-1},x_{t-h})\log\frac{p(y_{t}|y_{t-1},x_{t-h})}{p(y_{t}|y_{t-1})}.$$

The actual time lag δ at which variable X affects variable Y is defined as follows:

(9) $$\delta =arg\underset{h}{max}\left(SPO{TE}_{X\to Y}(h)\right).$$

Thus, the LSTE formulation becomes the following:

(10) $$LSTE_{X\to Y}=\sum p(y_{t},y_{t-1},x_{t-\delta })\log_{2}\frac{p(y_{t}|y_{t-1},x_{t-\delta })}{p(y_{t}|y_{t-1})}.$$

When δ=1, the LSTE reduces to the traditional TE, and Equation (10) can be rewritten in terms of entropy:

(11) $$\begin{array}{l} {LSTE}_{X\to Y}=-H\left(y_{t}|y_{t-1},x_{t-\delta }\right)+H(y_{t}|y_{t-1})=-H\left(y_{t},y_{t-1},x_{t-\delta }\right)+H\left(y_{t-1},x_{t-\delta }\right)\\ \;+H\left(y_{t},y_{t-1}\right)-H\left(y_{t-1}\right) \end{array}$$

### 3.2. Transfer Entropy Estimation

The calculation of transfer entropy (TE) involves numerous joint and conditional probability density estimations. Since the probability distributions of variables are typically unknown in real systems, it becomes necessary to approximate them. Non-parametric probability density estimation is widely adopted because it can handle arbitrary distribution forms without relying on specific assumptions. Common non-parametric methods include histograms, kernel density estimation (KDE) [30], and K-nearest neighbors (KNN) [31]. The histogram method is simple and intuitive, but it exhibits significant bias when applied to high-dimensional data. While KDE offers high accuracy, its computational complexity increases substantially with larger sample sizes and higher dimensions. Therefore, in this study, the KNN method is selected for non-parametric probability density estimation due to its balance between accuracy and efficiency.

According to Equation (11), estimating LSTE is equivalent to computing a combination of joint and marginal entropies. Each entropy term in Equation (11) can be estimated using the KNN method [31]. The pseudocode for LSTE estimation via KNN is shown in Algorithm 1.
**Algorithm 1** Estimation of $LSTE_{X\to Y}$ using KNN **Input:** Variable *X* and *Y,* embedding dimension d=l, embedding delay τ, number of neighbors k $\mathrm{Output}:\;LSTE_{X\to Y}$ N=length(X)−(d−1)× * τ−1 for i=1:d X_embed(:, i)=X((i−1)×τ+1: N+(i−1)×τ) Y_embed(:, i)=Y((i−1)×τ+1: N+(i−1)×τ) **End**
joint_space = [*Y*_future, *X*_embed, *Y*_embed]  % Construct joint space [nnidx, dists] = Knn_search(joint_space, k + 1); % Find local neighborhood using KNN maxdistV = dists(end,:) % Distance to the (k + 1)-th neighbor defines the hypersphere radius [nxz, nyz, nz] = point_estimation(*Y*_future, *X*_embed, *Y*_embed); % Estimate local point counts $LSTE_{X\to Y}$ = Digamma(nxz, nyz, nz) % Compute LSTE using Equation (12) $\mathrm{return}\;LSTE_{X\to Y}$


For a given number of neighbors k, *t* different spatial scales may arise across the terms in Equation (11) due to the varying dimensions of the associated variable spaces. Therefore, applying KNN directly to estimate each entropy term in Equation (11) can be problematic [31]. To address this issue, we adopt an improved TE estimation method that directly approximates the conditional mutual information and avoids explicit density estimation. The resulting expression for LSTE is as follows:

(12) $$LSTE_{X\to Y}=\psi \left(k\right)+\left\langle \psi \left(n_{y_{t-1}}+1\right)-\psi \left(n_{y_{t},y_{t-1}}+1\right)\right.{\left.-\psi \left(n_{y_{t-1},x_{t-\delta }}+1\right)\right\rangle }_{t},$$

where $\psi \left(\cdot \right)$ denotes the Digamma function and ${\langle \cdot \rangle }_{t}$ indicates averaging over all time steps. This approach improves computational efficiency and avoids the need to explicitly estimate sample probability densities.

### 3.3. Significance Test

In practice, transfer entropy values are often contaminated by noise or confounding effects, making direct inference unreliable. Therefore, we apply a surrogate-based significance test to determine whether a causal relationship exists between variables *X* and *Y*. The statistical significance of TE is assessed using a randomized test based on time-shifted surrogate sequences [32]. This approach disrupts the coupling between the driving and response variables, thereby creating a null distribution for comparison [33]. The pseudocode for the significance test is shown in Algorithm 2.
**Algorithm 2** Time-shifted substitution sequences**Input:** Variable X and Y, significance level α=0.05, number of surrogate M=100, time lag δ, causal flag *c*, random integer *d*_0_ Output:  p value, LSTE_original, δ = calculate_LSTE(X, Y)  % Compute original LSTE LSTE_surrogates = []  % Initialize surrogate array **for** i = 1 **to** M  % Generate surrogate data X_surr = shift_time_series(X, d_0_)  % Time shift X using Equation (13) LSTE_surr = calculate_LSTE(X_surr, Y) LSTE_surrogates = LSTE_surr % Store surrogate LSTE **end** Compute *p*_value according to Equation (14) if *p* value < *α*
**then** *c* = 1     % Causal relationship is statistically significant **end** **return** δ, *p* value

A random integer $d_{0}$ (where $d_{0}<n$, and n is the length of the time series) is generated, and the first $d_{0}$ samples of the driving variable are cyclically shifted to the end of the time series to construct a surrogate:

(13) $$\left\{x_{1},\ldots ,x_{d_{0}},x_{d_{0}+1},\ldots ,x_{n}\right\}\to {\left\{x_{d_{0}+1},\ldots ,x_{n},x_{1},\ldots ,x_{d_{0}}\right\}}^{\prime }$$

The null hypothesis $H_{0}$ assumes that there is no causal relationship between variables X and Y. The significance test compares the original transfer entropy value $q_{0}$ with those obtained from the *M* time shift surrogate series ($q_{1},\;\ldots ,\;q_{M}$). In this study, the significance level is set to α=0.05. The one-sided *p*-value is computed as follows:

(14) p=1−(i−0.326)/(M+1+0.348),

where i is the rank position of $q_{0}$ in ascending list of all M+1 values. If p<0.05, the null hypothesis is rejected, indicating a statistically significant causal relationship between X and Y.

### 3.4. Procedure of LSTE for Disturbance RCD

**Figure 1:**
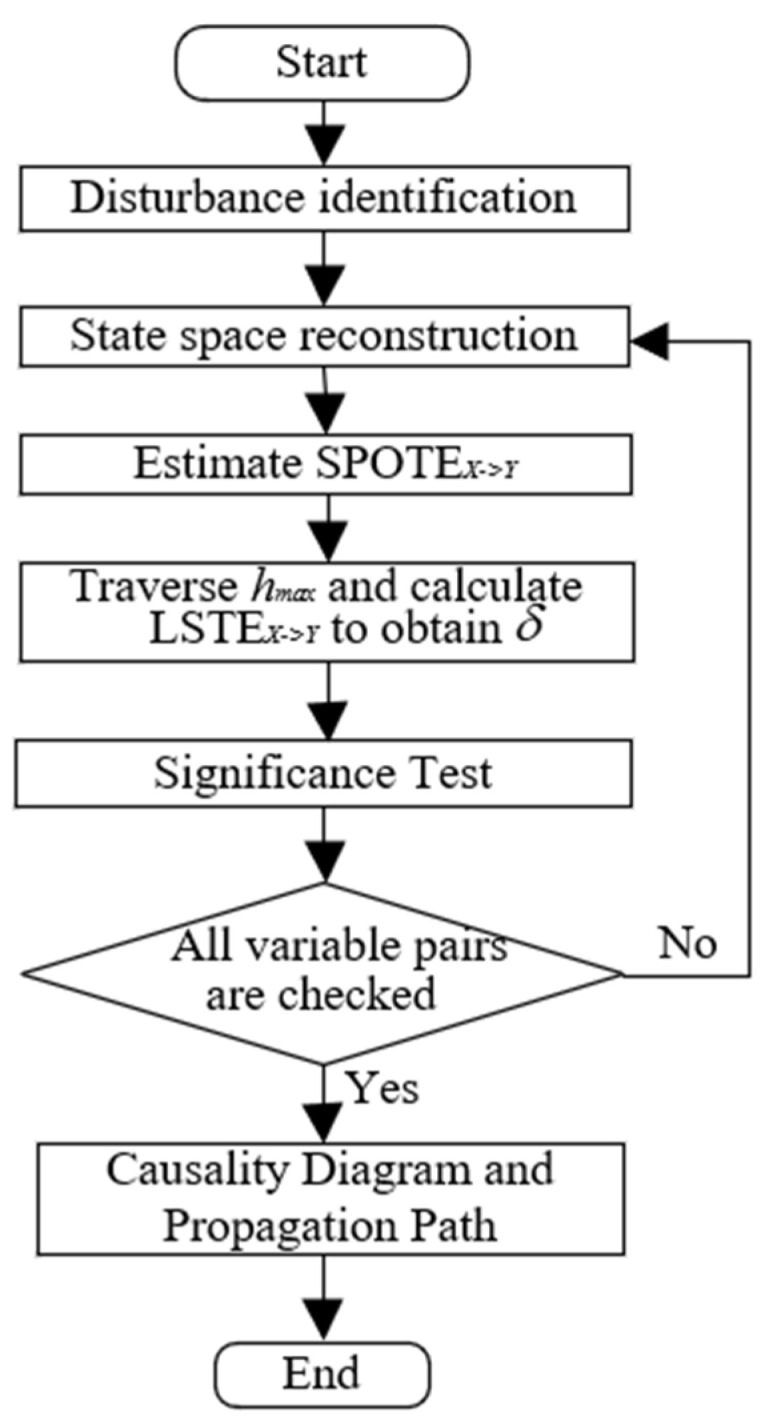
The flowchart of the LSTE-based RCD procedure.

Figure 1 illustrates the flowchart of the LSTE-based RCD framework. A detailed explanation of each step is provided below.

Before performing disturbance RCD, disturbance identification is conducted to filter out the most relevant variables from a large set of industrial process variables. Then, a pair of variables X and Y is selected, where X is the potential cause and Y is the effect. The linear and nonlinear trends contained in the time series are removed using MATLAB’s “detrend” function (MATLAB version: R2022a). The embedding dimension d and *l*, as well as the embedding delay τ, are selected using the Ragwitz criterion [34], which provides an automatic and statistically principled approach to embedding parameter selection, thereby eliminating the need for manual tuning. The past state of the system is then reconstructed via state space embedding of the scalar time series.

Next, the maximum delay lag $h_{max}$ is set and the value of ${SPOTE}_{X\to Y}$ under different h is estimated using the KNN algorithm, as shown in Equation (8). The optimal time lag δ and the corresponding lag-specific transfer entropy ${LSTE}_{X\to Y}$ are then determined according to Equations (9) and (10). A significance test is performed using the time shift surrogate method (Equation (14)). If p<0.05, a statistically significant causal relationship from X and Y is inferred; otherwise, no causal link is assumed.

The above steps are repeated for all variable pairs to comprehensively test causality throughout the system. Finally, the causality diagram is constructed based on the detected causal relationships and the disturbance propagation path is visualized.

When a variable is identified as the root cause of a disturbance in the causality diagram, it suggests that the system state represented by this variable (e.g., valve opening, feed rate) has exhibited abnormal fluctuations, or that a disturbance source exists in its neighboring components. Such diagnostic insight provides engineers with actionable guidance for timely mitigation and control interventions, reducing the impact on the overall system performance.

## 4. Case Studies

In this section, the numerical simulation, TEP, TPF, and a full-scale blast furnace line are used to illustrate the effectiveness of the proposed method. All experiments in this paper are conducted under the same environment (CPU i5 @ 1.60 GHz, RAM 12 GB).

### 4.1. Numerical Simulation

**Figure 2:**
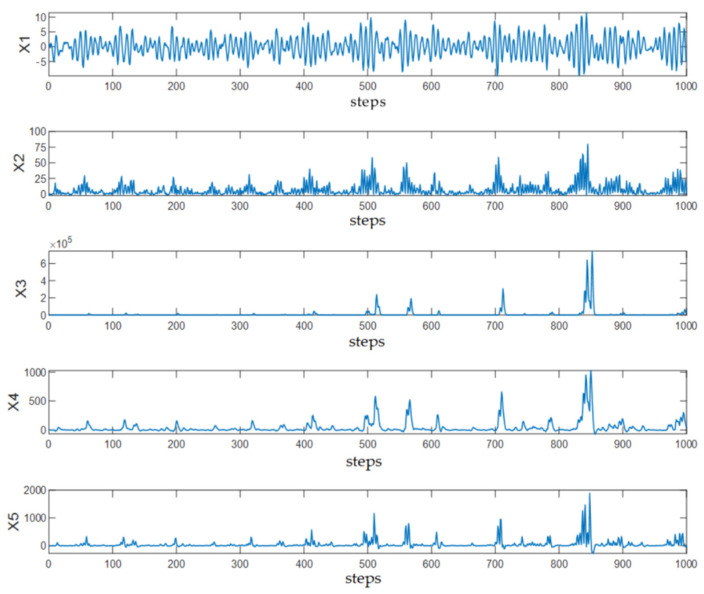
Trajectories of the simulated process variables.

A nonlinear simulation system containing five variables is designed in this study, with each variable having a length of n=1000. The system equation is as follows:

(15) $$\begin{array}{l} X_{1,t}=0.95\sqrt{2}X_{1,t-1}-0.9025X_{1,t-2}+\varepsilon_{1,t}\\ X_{2,t}=0.6X_{1,t-2}^{2}+\varepsilon_{2,t}\\ X_{3,t}=-0.2\sqrt{2}X_{1,t-3}+0.5\sqrt{2}X_{4,t-2}^{2}+\varepsilon_{3,t}\;\;\;\;\;\\ X_{4,t}=-0.5X_{1,t-2}^{2}+0.5\sqrt{2}X_{4,t-1}+0.25\sqrt{2}X_{5,t-1}+\varepsilon_{4,t}\\ X_{5,t}=-0.25\sqrt{2}X_{4,t-1}+0.5X_{5,t-1}+0.3X_{2,t-3}^{2}+\varepsilon_{5,t} \end{array}$$

where $\varepsilon_{i,t}\;(i=1,\ldots 5)$ represents independent Gaussian white noise with a mean of zero and unit standard deviation. The time series trajectories are shown in Figure 2.

As shown in Equation (15), $X_{1}$ is the root cause of the disturbance. The system contains seven causal paths in total, including linear causality ($X_{1}\to X_{3}$, $X_{4}\to X_{5}$) and nonlinear causality ($X_{1}\to X_{2}$, $X_{1}\to X_{4}$, $X_{2}\to X_{5}$, $X_{4}\to X_{3}$). In this study, TE [18] and PTE [20] are used as comparison algorithms to verify the effectiveness of the proposed method. A binary classification framework is adopted to evaluate the model’s diagnostic performance on the simulated system. In this context, the presence of a causal relationship is treated as a positive instance, and its absence as a negative instance. Based on the model’s prediction outcomes, we define four types of classification results. A true positive (TP) occurs when the model correctly identifies an existing causal relationship. A false positive (FP) arises when the model incorrectly predicts a causal relationship that does not exist. A true negative (TN) denotes the correct identification of the absence of causality. A false negative (FN) indicates a missed detection of an actual causal link.

Using these four outcomes, we compute four evaluation metrics to comprehensively assess model performance: accuracy, sensitivity (recall), specificity, and F1 score. Accuracy measures the proportion of all correctly classified instances (both TP and TN) among the total. Sensitivity represents the proportion of true causal relationships correctly identified. Specificity measures the proportion of non-causal pairs correctly rejected. The F1 score reflects the harmonic mean of precision and recall, offering a balanced indicator of model performance, particularly under class imbalance. The formulas for these metrics are given in Equations (16)–(19).

(16) $$Accuracy=\frac{TP+TN}{TP+TN+FP+FN}$$

(17) $$Sensitivity=\frac{TP}{TP+FN}$$

(18) $$Specificity=\frac{TN}{TN+FP}$$

(19) $$F1=\frac{2TP}{2TP+FP+FN}$$

**Figure 3:**
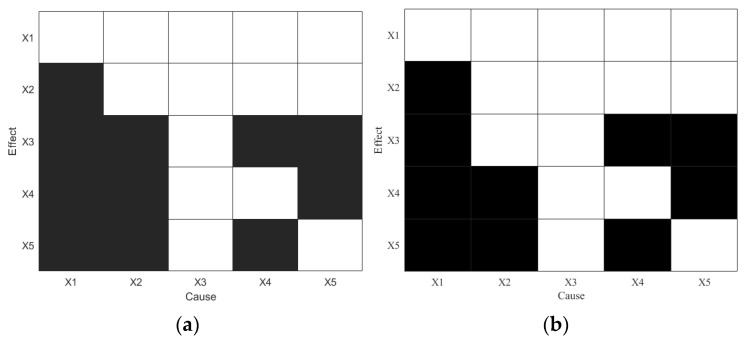
Causality diagrams for disturbance root diagnosis using (**a**) TE and (**b**) PTE in the numerical simulation.

Figure 3 presents the causality diagrams derived from the numerical simulation using TE and PTE, respectively. In these diagrams, the horizontal axis represents cause variables and the vertical axis represents effect variables. A black cell indicates a detected causal relationship between the corresponding variable pair, while a white cell indicates no causal link. If a variable’s entire row is white (no outgoing influence), but its column contains black cells (incoming influence), this suggests that the variable is the root cause of the disturbance.

As shown in Figure 3a, TE successfully identifies the true root cause variable $X_{1}$ and all seven true causal paths. However, it also incorrectly detects indirect causal links such as $X_{1}\to X_{5},\;X_{2}\to X_{4},\;X_{5}\to X_{3}$, and $X_{2}\to X_{3}$, which are considered false positives in this benchmark. Figure 3b illustrates the PTE result, which also correctly identifies the root cause variable, but with fewer false causal relationships. As shown in Table 1, PTE achieves a higher accuracy and F1 score than TE, suggesting improved precision in eliminating spurious causal paths.

**Figure 4:**
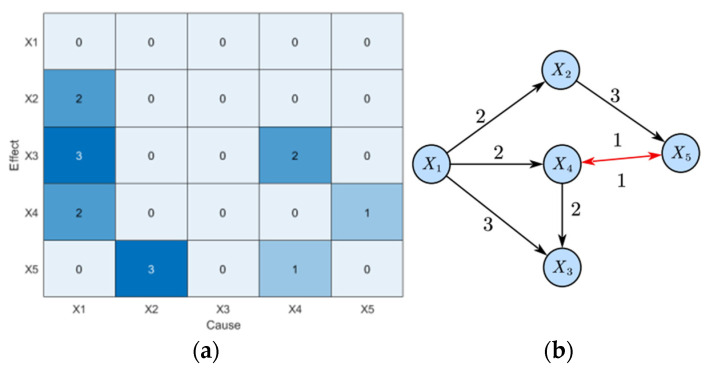
Diagnosis results of disturbance root in numerical simulation system (LSTE): (**a**) causality diagram and (**b**) propagation path.

Following this, the proposed LSTE method is applied to the same simulation system. Unlike TE and PTE, LSTE not only identifies whether a causal relationship exists between variable pairs, but also determines the corresponding time lag and the strength of the causal link. The results are summarized in Table 2 and visualized in Figure 4.

In Table 2, the values represent the estimated causal strengths, while the values in parentheses denote the corresponding *p*-values calculated using Equation (14). A causal relationship is considered statistically significant if *p* < 0.05. Values that meet this significance threshold are highlighted in bold.

The data in Table 2 corresponds to Figure 4a. Values in Figure 4a represent the time lag δ, indicating the delay at which the causal strength is highest. A value of 0 means no causal relationship. The corresponding causal strength values at that time lag are shown in Table 2.

As shown in the figure, the LSTE method correctly identifies the root cause $X_{1}$ and all true causal paths. Bidirectional causality is marked in red, and unidirectional causality is marked in black. The disturbance propagation path is: $X_{1}\overset{2}{⟶}X_{2}\overset{3}{⟶}X_{5}\overset{1}{↔}X_{4}\overset{2}{⟶}X_{3},$ which is consistent with the true disturbance propagation in the simulation system.

In addition, the robustness of LSTE under different signal-to-noise ratios (SNRs) was evaluated by adjusting the variance of the noise $\varepsilon_{i,t}$ in the numerical simulation experiment. The experimental results are summarized in Table 3.

As shown in Table 3, the performance of LSTE decreases as the SNR decreases. However, the root cause variable can still be identified at an SNR of –10 dB, indicating that LSTE possesses a certain degree of robustness.

### 4.2. Tennessee Eastman Process

#### 4.2.1. Brief Introduction

The TEP dataset used for experimental analysis was developed and made available by prior researchers. It has been described in detail in the literature [35] and is widely used in the process systems engineering community. The dataset can be accessed through the following link: https://web.mit.edu/braatzgroup/links.html (accessed on 23 June 2025). In each simulation run, the total process time is 48 h, with a sampling interval of 3 min, resulting in 960 data points per series. The first 160 data points correspond to steady-state (normal) operation, while the remaining 800 data points capture system behavior after the introduction of a disturbance.

The feed of the TE process consists of four gas reactants (A, C, D, E) and an inert protective gas (B). Three reaction products (G, H, F) are generated through four main chemical reactions. G and H are the desired products, while F is a by-product. The reaction equations are as follows:

(20) $$\begin{array}{cc} A+C+D\to G & \mathrm{Product}1\\ A+C+E\to H & \mathrm{Product}2\\ A+E\to F & \;\mathrm{By}\text{-}\mathrm{product}1\\ 3D\to 2F & \;\mathrm{By}\text{-}\mathrm{product}2 \end{array}$$

As described in Appendix A, the TEP system primarily consists of five units: a stirred reactor, stripping tower, product separator, compressor, and condenser. The platform provides 52 diagnostic variables, including 11 control variables and 41 process variables.

TEP includes 21 preset disturbance scenarios, such as step changes, valve-related disturbances, and increases in process variability. For a detailed description of the TEP variables and disturbance scenarios, please refer to references [35,36].

#### 4.2.2. IDV 5

Disturbance IDV5 is a step disturbance caused by a temperature change at the condenser’s cooling water inlet. When the disturbance occurs, it first affects process variables related to the separator, such as the separator temperature ($X_{11}$) and the separator cooling water outlet temperature ($X_{22}$), due to the direct connection between the condenser and the separator. The disturbance then propagates to other units, affecting variables such as the reactor pressure ($X_{7}$), stripper pressure ($X_{16}$), and compressor power ($X_{20}$). To compensate for the disturbance, the feedback controller adjusts the condenser cooling water flow rate ($X_{52}$). After the process reaches a new steady state, variable $X_{52}$ does not return to its original value, whereas the other variables return to normal. Based on this behavior, $X_{52}$ is identified as the root cause variable of IDV5.

**Figure 5:**
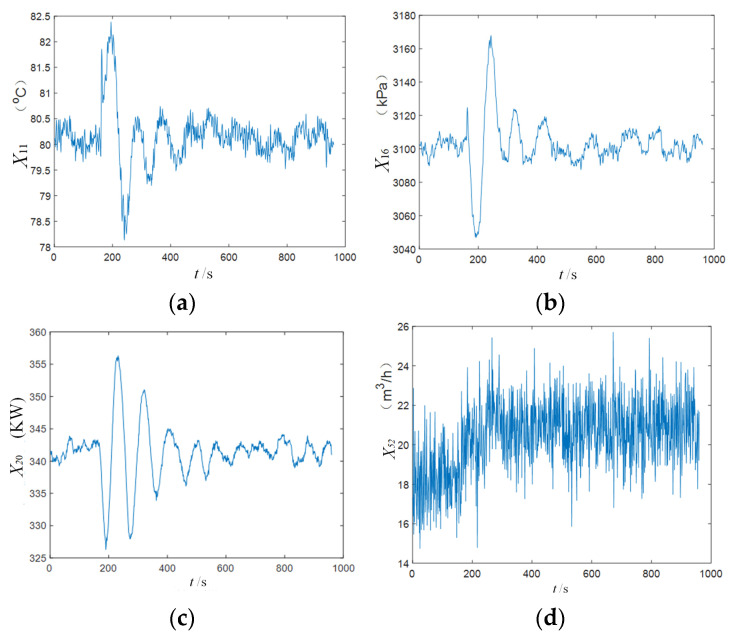
The change track of some variables (IDV5): (**a**) variable $X_{11}$ (temperature), (**b**) variable $X_{16}$ (flow), (**c**) variable $X_{20}$ (temperature), and (**d**) variable $X_{52}$ (flow).

The time series trends of selected variables after the occurrence of IDV5 are shown in Figure 5. It can be observed that each variable exhibits a step change immediately following the disturbance, followed by damped oscillations that eventually stabilize.

Before applying TE, it is necessary to eliminate irrelevant variables and retain only those most associated with the disturbance, i.e., perform disturbance identification [37,38]. This study directly adopts the variable selection results from reference [37], yielding the set $\left\{X_{7},X_{11},X_{16},X_{19},X_{20},X_{22},X_{50},X_{52}\right\}$.

**Figure 6:**
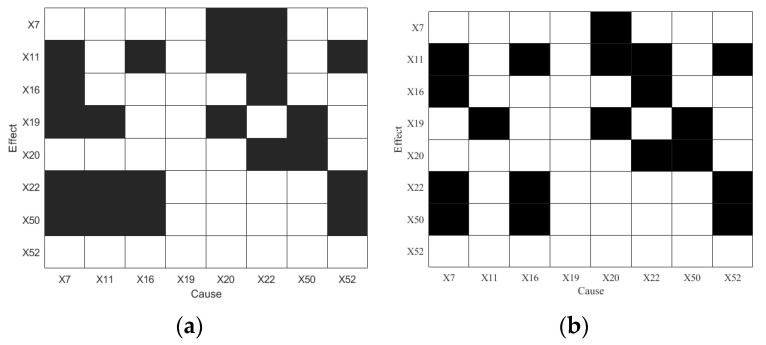
Diagnosis results of IDV5: (**a**) TE and (**b**) PTE.

The second step is data segment selection. In this experiment, the starting point is set to the moment the disturbance occurs, and the segment length is 800, corresponding to time steps 161–960. The embedding dimension is set to d=l=4, and the embedding delay is τ=1. The TE and PTE are used to conduct RCD of IDV5, and the diagnosis results are shown in Figure 6.

As shown in Figure 6a, variable $X_{52}$ is identified as having a causal effect on $X_{11}$, $X_{22}$, and $X_{50}$, and is not influenced by other variables, correctly indicating $X_{52}$ as the disturbance root cause of IDV5. However, TE also detects numerous cyclic causal relationships (e.g., ${X_{11}↔X}_{22}$, ${X_{16}↔X}_{22}$), which obscure the true disturbance propagation path. Similarly, while PTE also identifies *X*_52_ as the root cause, it still exhibits bidirectional causal links, such as ${X_{16}↔X}_{22}$.

Next, LSTE is applied to diagnose the root cause of IDV5. The maximum lag is set to $h_{max}=10$. The causal strengths among the selected variables, along with their statistical significance, are reported in Table 4, and the corresponding causality diagram is shown $X_{11}$ to $X_{20}$ occurring after nine sampling intervals.

**Figure 7:**
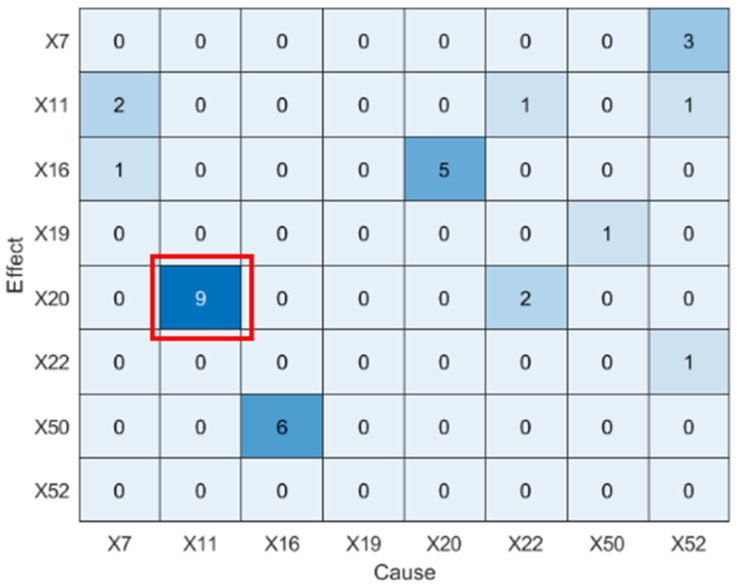
Causality diagram of IDV5 (LSTE).

In Figure 7. Statistically significant values (*p* < 0.05) are highlighted in bold. In addition, in the causality diagram, the x-axis and y-axis represent the same set of variables (i.e., disturbance variables $\left\{X_{7},X_{11},X_{16},X_{19},X_{20},X_{22},X_{50},X_{52}\right\}$), with the x-axis denoting cause variables and the y-axis denoting effect variables. Each color block indicates the presence and strength of information transfer between variable pairs at specific time lags. The red box highlights a detected directional influence from $X_{11}$ to $X_{20}$ occurring after 9 sampling intervals.

**Figure 8:**
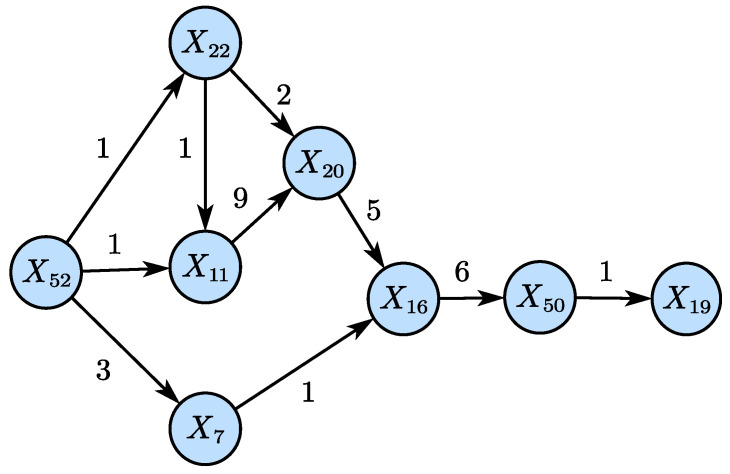
Propagation path of IDV5 (LSTE).

Figure 8 illustrates the disturbance propagation path derived from Figure 7, highlighting the time delays between causally related variables. When the disturbance occurs, variable $X_{52}$ affects $X_{11}$ and $X_{22}$ after one time delay, meaning that the adjustment in the condenser cooling water flow ($X_{52}$) influences the separator temperature ($X_{11}$) and the separator cooling water outlet temperature ($X_{22}$) within 3 min. Subsequently, after 9 min, it influences the reactor pressure ($X_{7}$).

The propagation path and time lags obtained from LSTE are consistent with process mechanism analysis. Comparing Figure 6 and Figure 8 reveals that the LSTE-based RCD method not only correctly identifies the disturbance root cause, but also determines the transmission time between variables, demonstrating the advantages of LSTE over traditional methods.

#### 4.2.3. IDV 8

Disturbance IDV8 is caused by random fluctuations in the feed ratios of components A, B, and C in Stream 4. When the disturbance occurs, it directly affects the corresponding measurements in Stream 4. The total feed rate controller ($X_{45}$) compensates for the disturbance by adjusting the total feed rate ($X_{4}$) in Stream 4 to maintain the setpoint. Since Stream 4 primarily contributes residual reactants to Stream 10, which are then recycled back to the reactor via Stream 5, the feed composition in the reactor changes, disrupting the dynamic balance of the chemical reactions. During this circulation, variables such as the reactor pressure ($X_{7}$), separator pressure ($X_{13}$), and the feed amount of component A in Stream 1 ($X_{1}$) are also affected. Based on this mechanism analysis, *X*_4_ and *X*_45_ are identified as the root variables of IDV8.

**Figure 9:**
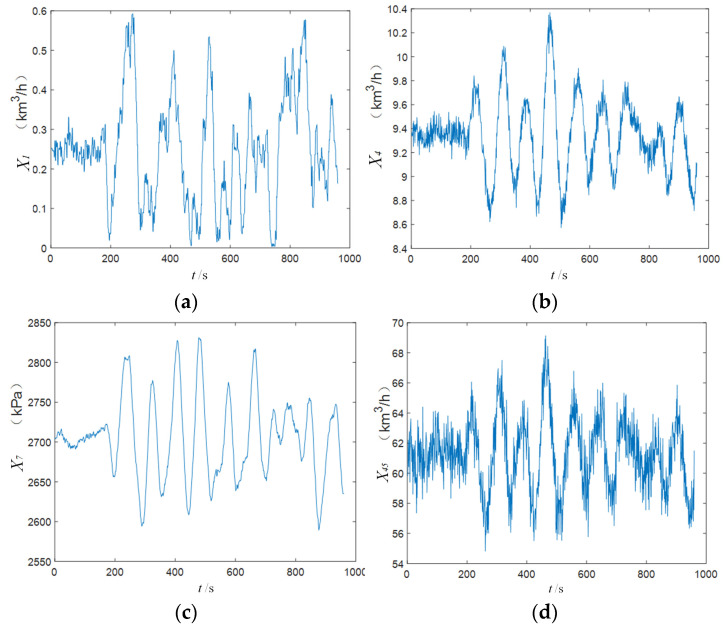
Change track of some variables (IDV8): (**a**) variable $X_{1}$ (feed amount), (**b**) variable $X_{4}$ (feed amount), (**c**) variable $X_{7}$ (pressure), and (**d**) variable $X_{45}$ (feed amount).

The time series changes in several relevant variables after the disturbance are shown in Figure 9. It can be observed that the values of the variables begin to change after time point 160, indicating the moment at which the disturbance occurs. The resulting fluctuations in the affected variables show stable periodic behavior, suggesting that IDV8 is a stable disturbance.

As with other cases, disturbance identification is necessary prior to performing root cause diagnosis (RCD) for IDV8. Based on the principal component analysis (PCA)-based contribution diagram method, the identified disturbance-related variables are $\left\{X_{1},X_{4},X_{7},X_{13},X_{16},X_{20},X_{21},X_{45},X_{46}\right\}$. This study uses the 800 data points following the disturbance (i.e., samples 161–960) as the experimental dataset. The embedding dimension is set to d=l=3 and the embedding delay is τ=1.

**Figure 10:**
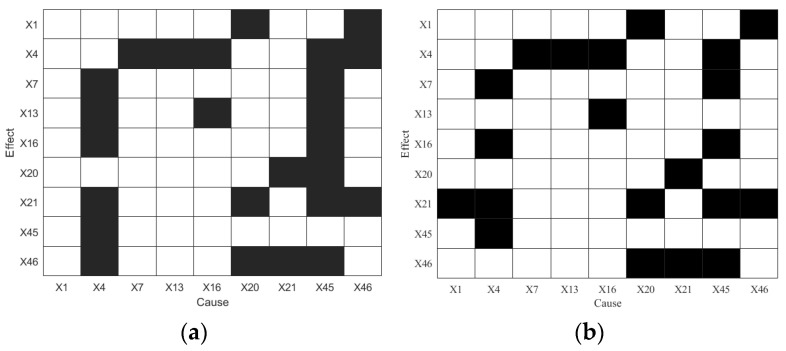
Diagnosis results of IDV8: (**a**) TE and (**b**) PTE.

TE and PTE are applied to diagnose the root cause of IDV8, and the results are shown in Figure 10. As shown in Figure 10a, variables *X*_4_ and *X*_45_ influence each other, but *X*_45_ is only influenced by *X*_4_, indicating that *X*_45_ is the root cause of IDV8. Both *X*_4_ and *X*_45_ affect multiple variables such as *X*_7_ and *X*_13_; however, since those variables also have causal effects on *X*_4_, *X*_4_ cannot be conclusively identified as a root cause. In Figure 10b, the number of indirect causalities is reduced, yet some bidirectional causalities remain (e.g., *X*_4_ ↔ *X*_7_).

**Figure 11:**
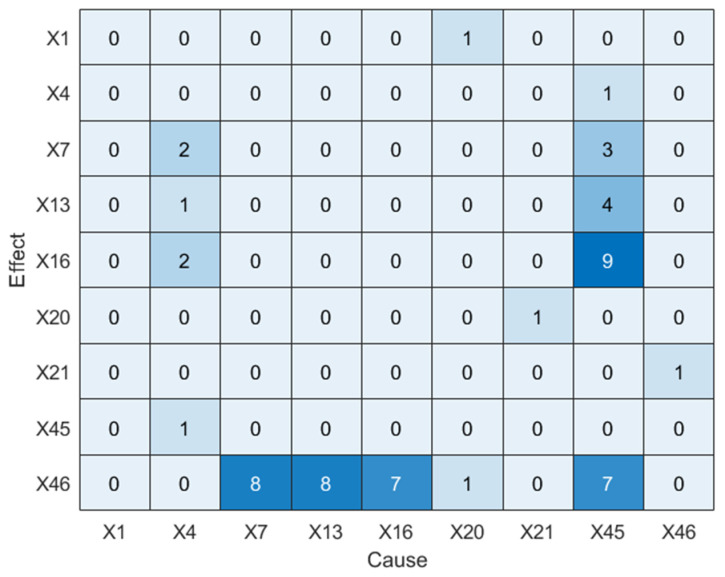
Causality diagram of IDV8 (LSTE).

Next, LSTE is applied to diagnose the root cause. The causal strengths among IDV8-related variables are listed in Table 5, and the corresponding causality diagram is shown in Figure 11. Statistically significant values (*p* < 0.05) are highlighted in bold.

**Figure 12:**
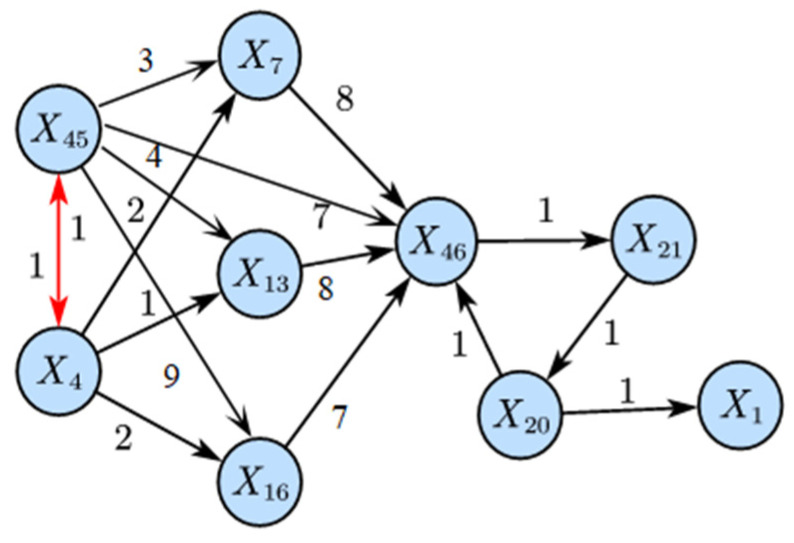
Propagation path of IDV8 (LSTE).

As seen in Figure 11, LSTE correctly identifies *X*_45_ as the root cause variable. The disturbance propagation path derived from this result is shown in Figure 12, which also reveals the time lag of causal transmission between variables.

Specifically, *X*_45_ affects *X*_4_ within 3 min. This is expected, since *X*_45_ (total feed controller) and *X*_4_ (total feed flow) are control and measurement variables of the same stream and are physically linked. Thus, the disturbance can be sensed almost simultaneously. Subsequently, after 6 min, the disturbance propagated to the reactor, disrupting the material balance and affecting the reactor pressure (*X*_7_) after 9 min. The impact then extended to the separator pressure (*X*_13_) after 12 min and the compressor re-circulation valve opening (*X*_46_) after 21 min. Finally, the disturbance reached the stripper, influencing the stripper pressure (*X*_16_) after 27 min.

By comparing Figure 10 and Figure 11, it is evident that the LSTE-based RCD method not only accurately identifies the root cause of the disturbance, but also captures the transmission time lags between disturbance-related variables, demonstrating the superiority of LSTE.

### 4.3. Three-Phase Flow Process

#### 4.3.1. Brief Introduction

The TPF dataset was collected by researchers at Cranfield University using a lab-scale experimental setup. Details of the experimental platform and data acquisition process (sampling frequency: 1 Hz) are provided in [39] and Appendix B. This setup mimics industrial Three-Phase Flow separation systems and simulates real-world disturbance scenarios, such as congestion, operational errors, and unconventional operating conditions. The dataset is publicly available at the following link: https://github.com/ShiningLLH/ThreePhaseFlow_Ag-MRDCVA (accessed on 23 June 2025).

The test area consists of pipes of various apertures and geometries, supplying air, water, and oil—individually and in mixtures—at controlled flow rates. The multiphase mixture is then separated into a horizontal three-phase separator (GS500) with a volume of 11 m^3^. Air is discharged into the atmosphere, while emulsions of oil and water are separated into their respective condensers (CW500 and CO500, each with a capacity of approximately 1.5 m^3^) before returning to their designated tanks (T200 for water and T100 for oil). For further details regarding the TPF variables and disturbances, refer to [39].

#### 4.3.2. Disturbance 5

Disturbance 5 (Slugging conditions) is selected as a validation case to evaluate the effectiveness of the proposed method. Slugging is an instantaneous phenomenon [40] that arises in multiphase flow risers when gas–liquid velocities are relatively low. In offshore oil production systems, when multiphase hydrocarbon fluids travel through long seabed pipelines from oil fields to offshore platforms, substantial pressure and flow fluctuations may occur, potentially damaging downstream equipment. Slugging conditions cause significant fluctuations in key process variables—such as pressure, flow rate, and density—at both the top and bottom of risers. Based on this mechanism analysis, the flow rate at the top riser (*X*_10_) and density at the top riser (*X*_13_) are identified as the root cause variables [41].

**Figure 13:**
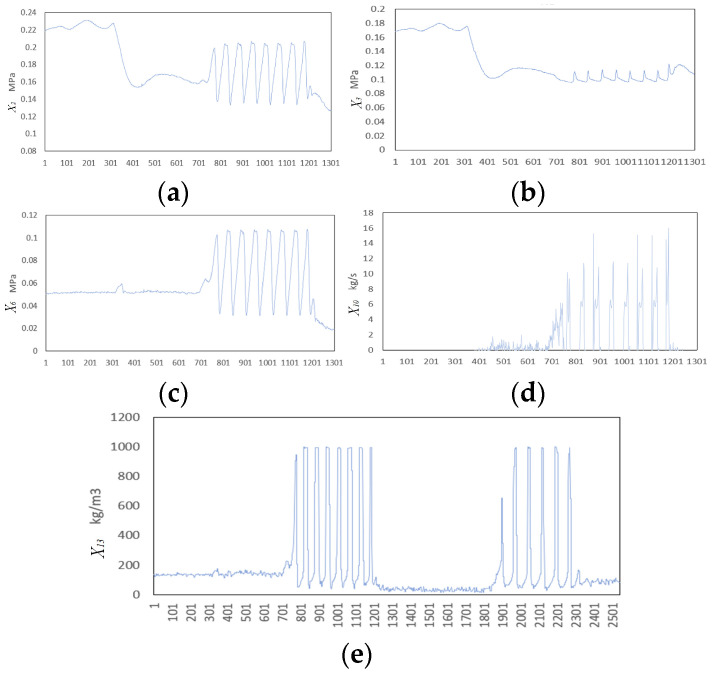
Trajectories of selected variables under Disturbance 5: (**a**) $X_{2}$, (**b**) $X_{3}$, (**c**) $X_{6}$, (**d**) $X_{10}$, and (**e**) $X_{13}.$.

**Figure 14:**
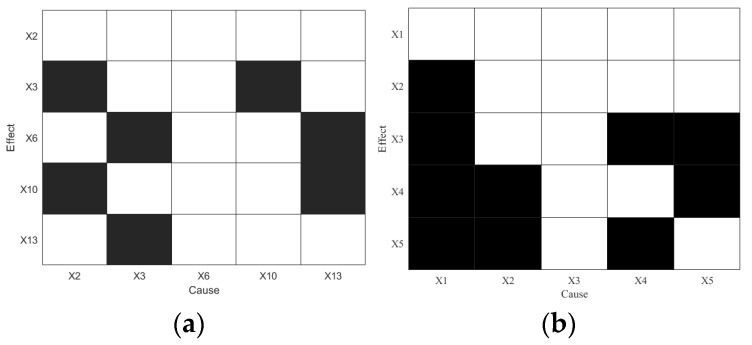
Diagnosis results of Disturbance 5: (**a**) TE and (**b**) PTE.

After the onset of Disturbance 5, the changing trends in selected variables are shown in Figure 13. The disturbance spans 2541 s, beginning at 686 s and ending at 1172 s. This study adopts the variable selection results from [41], which include $\left\{X_{2},X_{3},X_{6},X_{10},X_{13}\right\}$. A total of 486 data points (1 Hz sampling) covering the entire disturbance duration are used, starting from the disturbance onset. The embedding dimension is set to *d* = *l* = 3, and the embedding delay is *τ* = 1. Traditional TE is first applied for RCD, and the results are shown in Figure 14a.

**Figure 15:**
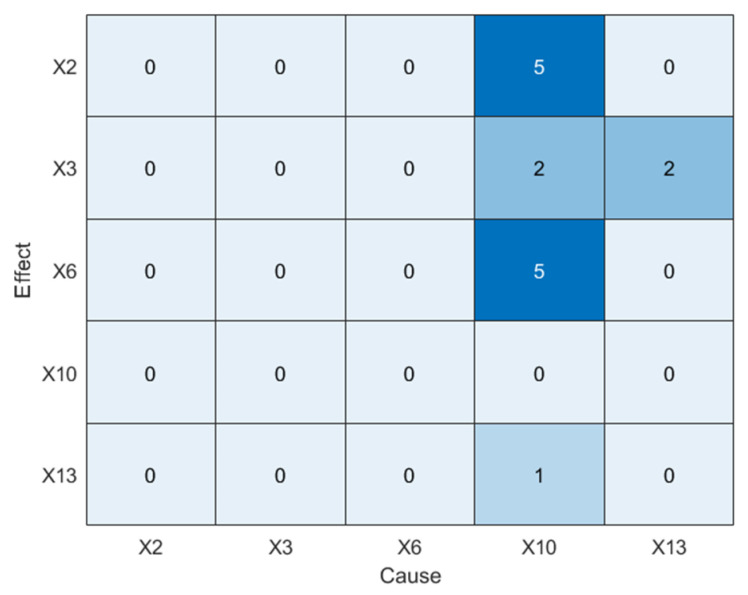
Causality diagram of Disturbance 5 (LSTE).

As shown in Figure 14a, TE incorrectly identifies *X_2_* as the root cause, which contradicts the mechanism-based analysis, indicating that TE fails to accurately identify the true root cause. Similarly, PTE also identifies incorrect root variables and exhibits several spurious causal relationships. Next, the LSTE method is applied, with the maximum lag delay set to $h_{max}=10$. The causal strengths and corresponding statistical significance results are summarized in Table 6 and visualized in Figure 15.

**Figure 16:**
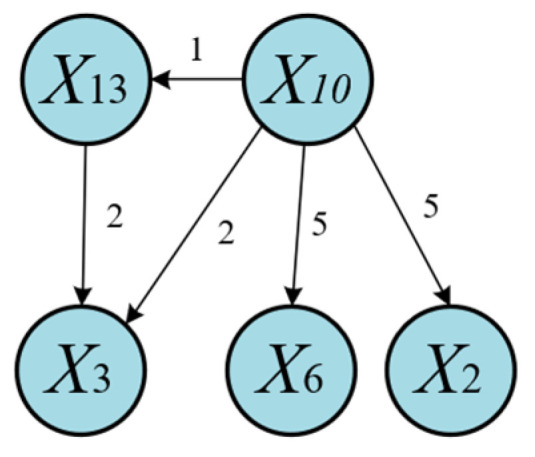
Propagation path of Disturbance 5 (LSTE).

As shown in Figure 15, LSTE correctly identifies *X*_10_ as the root cause variable, with causal effects on other variables. Figure 16 illustrates the disturbance propagation path derived from Figure 15 and highlights the time lags associated with causal transmission.

Specifically, *X*_10_ affects *X*_13_ within 1 s, which is expected given the strong physical coupling between the flow rate and density at the top riser. These two variables reflect the system’s state at the same spatial location. After 2 s, the disturbance propagates to the pressure at the top of the riser (*X*_3_). Finally, the disturbance reaches the bottom of the riser, affecting *X*_2_ (bottom pressure) and *X*_6_ (differential pressure) after 5 s. The longer transmission time is attributed to the physical distance along the riser between *X*_10_ and *X*_2_.

### 4.4. Blast Furnace Ironmaking Process

#### 4.4.1. Brief Introduction

The blast furnace ironmaking process is a complex, high-temperature system involving the transfer of matter and energy among four distinct phases: gas (coal gas), solid (ore, coke, and flux), liquid (slag and molten iron), and powder (coal powder, etc.) [42,43]. It represents one of the most intricate reaction mechanisms in the field of chemical metallurgy. Investigating the time-lagged causal relationships between key operating variables and the molten iron temperature (PT) provides critical decision support for on-site operations. In the following experiment, data from two days of blast furnace operation were analyzed separately. With a sampling interval of 5 min, each dataset consists of 288 data points. The ironmaking data used in this study were obtained from an industrial project led by the authors. The dataset is proprietary, and its use has been explicitly approved by the collaborating company for research and publication purposes.

#### 4.4.2. Results Analysis

**Figure 17:**
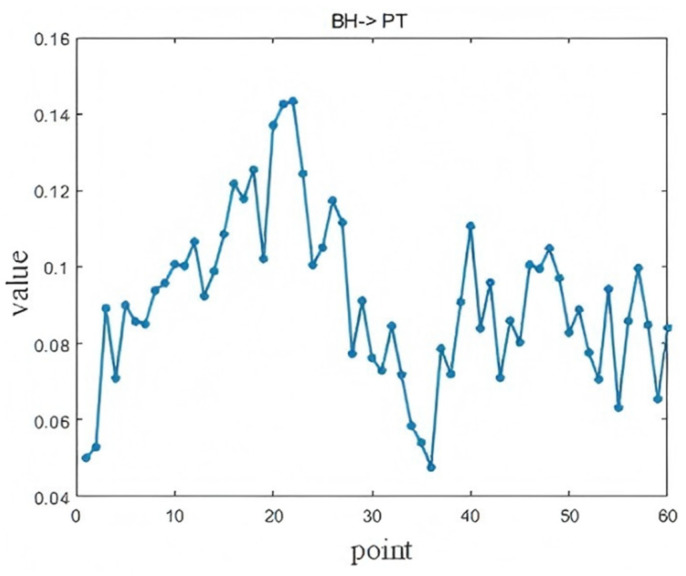
Diagnosis results of BH → PT.

**Figure 18:**
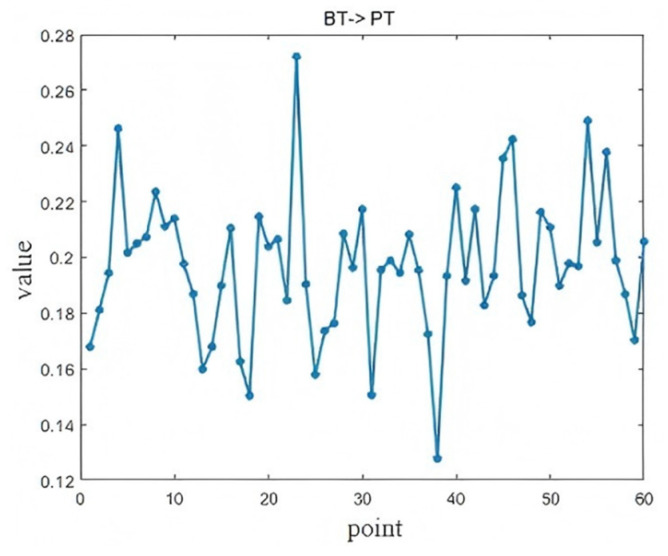
Diagnosis results of BT → PT.

**Figure 19:**
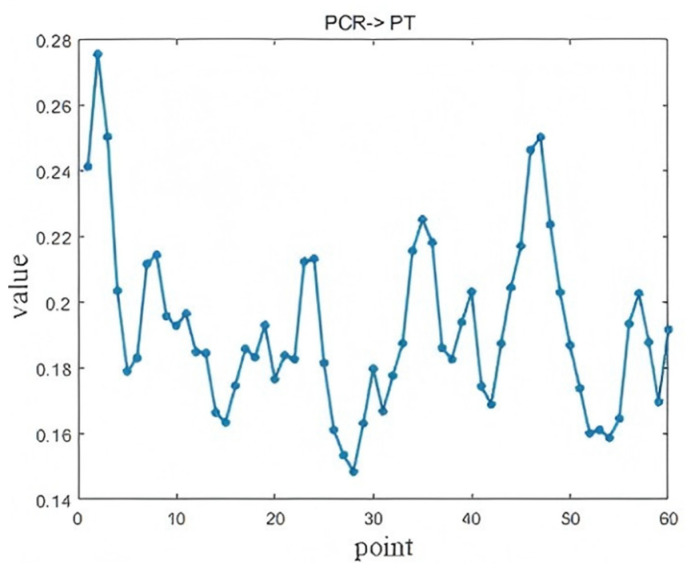
Diagnosis results of PCR → PT.

The causal time lag analysis results between three key operating variables—Blast Temperature (BT), Blast Humidity (BH), and Pulverized Coal Ratio (PCR)—and the Pig iron Temperature (PT) are presented in Figure 17, Figure 18, and Figure 19, respectively. The maximum LSTE values identified in each case pass the statistical significance test (*p* < 0.05).

As shown in Figure 17, the effect of BH on the PT is most prominent at the 22nd lag point, corresponding to a time lag of 110 min (*p* = 0.0217). BH represents the moisture content of the gas entering the furnace, and directly affects combustion and heat transfer. As the humidity increases, more heat is required for vaporization, increasing the furnace’s thermal load. Since the moisture must travel through several complex zones—such as the combustion and reduction zones—before affecting the molten iron, a time lag is introduced. Understanding this lag allows operators to anticipate how changes in humidity will impact the furnace temperature, and to prevent sharp fluctuations caused by excessive or insufficient moisture.

In Figure 18, the influence of BT on the PT peaks at the 23rd lag point, approximately 115 min after the change (*p* = 0.0034). BT is a critical parameter in the combustion and reduction reactions within the furnace. Higher air temperatures improve combustion efficiency, increasing heat generation and thereby raising the PT. The observed delay is reasonable, as the elevated air temperature must pass through multiple reaction and heat transfer zones before impacting the molten iron. Recognizing this lag helps operators to fine-tune BT adjustments more effectively and avoid overcorrections.

As shown in Figure 19, the PCR affects the PT at two distinct lag points. The first effect appears at the 2nd lag (approximately 10 min, *p* = 0.0072), attributed to the rapid heat absorption and decomposition of hydrocarbons in the injected pulverized coal, which causes short-term fluctuations in the PT. The second, more pronounced effect occurs at the 47th lag (approximately 235 min). This longer-term influence results from the slow ascent and chemical interaction of reducing gases (CO + H_2_) generated by coal combustion. These gases rise through the furnace, reacting with iron ore and releasing heat, thus producing a delayed impact on the molten iron temperature.

The above causal time lag analysis has been reviewed and validated by blast furnace ironmaking experts. These results highlight the heterogeneous influence of different variables on the PT, reflecting the complexity and temporal dynamics of heat and mass transfer within the blast furnace. Since the effects of process variables on the PT do not occur simultaneously, analyzing their time lag characteristics enables more effective predictive control, helps mitigate sharp temperature fluctuations, and improves system stability and production efficiency. Moreover, understanding these time lag relationships allows for the selection of appropriate historical data as model inputs, ensuring temporal consistency in PT prediction models and enhancing their overall accuracy.

## 5. Conclusions

Although various TE-based RCD methods have been studied, important limitations remain. This work proposes an LSTE approach that simultaneously estimates both the time delay and causal strength between process variables. The proposed algorithm is applicable to multivariate time delay causal analysis and root cause diagnosis in industrial systems. All computations were performed on a Windows-based system equipped with an Intel i5 processor (1.60 GHz) and 12 GB of RAM. The algorithm was implemented in MATLAB (MATLAB version: R2022a). The method was validated on a numerical simulation and two industrial benchmarks—the TEP and TPF systems. In all cases, LSTE provided meaningful estimates of delay and causal strength, enabling the accurate identification of the root cause variable. The results aligned with known process mechanisms, enhanced understanding of disturbance propagation, and offered actionable insights for plant operators. The framework was further applied to analyze time-lagged causal relationships between the PT and key operating variables in a blast furnace, providing practical guidance for real-time operations.

However, transfer entropy-based methods, including the proposed LSTE approach, are computationally intensive—particularly when applied to medium- or large-scale systems with high-dimensional variable spaces. This creates challenges for real-time implementation. To address scalability, a candidate variable selection step is typically required prior to root cause diagnosis. By identifying a smaller subset of critical variables, the computational burden can be substantially reduced without compromising diagnostic performance. As this study focuses specifically on root cause diagnosis (RCD), the candidate set identification step is not elaborated upon here. Readers interested in this aspect can refer to our prior work [44] for more details on candidate set identification strategies.

It is also worth noting that the proposed algorithm focuses on root cause diagnosis under the assumption of a single disturbance. However, in real-world industrial processes, multiple disturbances may occur simultaneously. Addressing this challenge presents a promising direction for future research, where more sophisticated algorithms capable of handling concurrent disturbances will be investigated. While this study focuses on the development and validation of the LSTE-based RCD method under stationary conditions, we acknowledge that non-stationarity—a prevalent feature in industrial systems—poses significant challenges. Designing RCD algorithms that can adapt to time-varying dynamics will be an important avenue for future work. Furthermore, high-frequency data introduces additional complications, including increased measurement noise and transient fluctuations, which may lead to false positive causal detections in TE-based methods. To partially mitigate this, we evaluated the robustness of LSTE under varying signal-to-noise ratio (SNR) levels. Nonetheless, the computational burden associated with large high-frequency datasets remains a challenge, and future research will also focus on scalable and noise-robust algorithmic improvements.

## Figures and Tables

**Table 1:** Performance comparison of different algorithms.

	Accuracy	Sensitivity	Specificity	F1 Score	Root Cause
TE	0.84	0.64	1.00	0.78	Yes
PTE	0.88	0.70	1.00	0.82	Yes
LSTE	1.00	1.00	1.00	1.00	Yes

**Table 2:** ${LSTE}_{X_{i}\to X_{j}}$ values and significance between simulation system variables. (The bold fonts in the table indicate causal values that pass the significance test).

	**To From**	$X_{1}$	$X_{2}$	$X_{3}$	$X_{4}$	$X_{5}$
**To**						
$X_{1}$		0	0.0009 (0.213)	0.0013 (0.154)	0.0021 (0.173)	0.0023 (0.324)
$X_{2}$		**0.407** (0.016)	0	0.001 (0.227)	0.007 (0.632)	0.002 (0.542)
$X_{3}$		**0.121** (0.022)	0.038 (0.367)	0	**0.067** (0.004)	0.081(0.617)
$X_{4}$		**0.107** (0.005)	0.152 (0.331)	0.003 (0.514)	0	**0.321** (0.024)
$X_{5}$		0.136 (0.604)	**0.226** (0.017)	0.004 (0.324)	**0.096** (0.041)	0

**Table 3:** Results of LSTE robustness testing.

	Accuracy	Sensitivity	Specificity	F1 Score	Root Cause
−10 dB	0.68	0.4545	0.8571	0.5556	Yes
−5 dB	0.60	0.3636	0.7857	0.4444	No
5 dB	0.64	0.4	0.8	0.4706	No
10 dB	0.64	0.4167	0.8462	0.5263	No

**Table 4:** Causal strength between IDV5-related variables (LSTE).

	**From**	$X_{7}$	$X_{11}$	$X_{16}$	$X_{19}$	$X_{20}$	$X_{22}$	$X_{50}$	$X_{52}$
**To**									
$X_{7}$		0	0.061 (0.4832)	0.125 (0.3743)	0.123 (0.5314)	0.135 (0.4251)	0.032 (0.3241)	0.111 (0.2148)	**0.007** (0.0081)
$X_{11}$		**0.161** (0.0032)	0	0.129 (0.4126)	0.050 (0.6143)	0.106 (0.6214)	**0.092** (0.0062)	0.046 (0.4263)	**0.017** (0.0072)
$X_{16}$		**0.171** (0.0067)	0.072 (0.3284)	0	0.121 (0.3281)	**0.139** (0.0071)	0.039 (0.5126)	0.109 (0.6327)	0.006 (0.4237)
$X_{19}$		0.150 (0.3625)	0.068 (0.6482)	0.142 (0.3926)	0	0.134 (0.0852)	0.008 (0.4571)	**0.169** **(0.0063)**	−0.001 (0.3247)
$X_{20}$		0.146 (0.6236)	**0.092** (0.0041)	0.139 (0.6128)	0.115 (0.2651)	0	**0.041** (0.0071)	0.116 (0.4351)	−0.003 (0.2853)
$X_{22}$		0.063 (0.2317)	0.087 (0.5132)	0.077 (0.3625)	0.043 (0.3184)	0.054 (0.4122)	0	0.043 (0.3418)	**0.026** (0.0073)
$X_{50}$		0.148 (0.1436)	0.066 (0.1326)	**0.153** (0.0036)	0.112 (0.1627)	0.130 (0.3124)	0.008 (0.3589)	0	0.006 (0.4827)
$X_{52}$		0.015 (0.2436)	0.033 (0.2135)	0.017 (0.2953)	0.015 (0.3846)	0.009 (0.5246)	0.021 (0.6251)	0.016 (0.4126)	0

**Table 5:** Causal strength matrix for IDV8 (LSTE).

	**From**	$X_{1}$	$X_{4}$	$X_{7}$	$X_{13}$	$X_{16}$	$X_{20}$	$X_{21}$	$X_{45}$	$X_{46}$
**To**										
$X_{1}$		0	0.036 (0.6214)	0.042 (0.8412)	0.039 (7243)	0.042 (0.4813)	**0.056** (0.0051)	0.039 (0.3177)	0.007 (0.3625)	0.042 (0.5134)
$X_{4}$		0.051 (0.1463)	0	0.114 (0.3146)	0.111 (0.4381)	0.117 (0.2546)	0.060 (0.3244)	0.067 (0.3242)	**0.068** (0.0041)	0.095 (0.4623)
$X_{7}$		0.071 (0.2641)	**0.151** (0.0072)	0	0.182 (0.6239)	0.189 (0.3581)	0.099 (0.3471)	0.058 (0.5132)	**0.096** (0.6423)	0.116 (0.6231)
$X_{13}$		0.074 (0.5162)	**0.162** (0.0016)	0.189 (0.2314)	0	0.194 (0.2163)	0.103 (0.2534)	0.062 (0.4326)	**0.094** (0.0072)	0.121 (0.7122)
$X_{16}$		0.079 (0.4653)	**0.115** (0.0324)	0.131 (0.3812)	0.132 (0.6124)	0	0.091 (0.3152)	0.056 (0.3421)	**0.078** (0.0042)	0.099 (0.3251)
$X_{20}$		0.064 (0.6438)	0.070 (0.6312)	0.115 (0.2147)	0.114 (0.4231)	0.122 (0.2731)	0	**0.108** (0.0126)	0.031 (0.4631)	0.118 (0.6211)
$X_{21}$		0.039 (0.3512)	0.097 (0.1843)	0.084 (0.1762)	0.083 (0.3621)	0.075 (0.7211)	0.078 (0.6427)	0	0.045 (0.4352)	**0.079** (0.0041)
$X_{45}$		0.013 (0.2315)	**0.074** (0.0037)	0.061 (0.0832)	0.062 (0.2573)	0.045 (0.2372)	0.015 (0.2144)	0.027 (0.5231)	0	0.031 (0.3214)
$X_{46}$		0.061 (0.3415)	0.094 (0.5236)	**0.139** (0.0483)	**0.137** (0.0037)	**0.142** (0.0041)	**0.139** (0.0034)	0.133 (0.4326)	**0.056** (0.0034)	0

**Table 6:** Causal strength matrix for Disturbance 5 (LSTE).

	**From**	$X_{1}$	$X_{4}$	$X_{7}$	$X_{13}$	$X_{16}$
**To**						
$X_{1}$		0	0.163 (0.4326)	0.032 (0.3251)	**0.037** (0.0023)	0.053 (0.3214)
$X_{4}$		0.141 (0.2341)	0	0.053 (0.4312)	**0.113** (0.0041)	**0.178** (0.0057)
$X_{7}$		0.115 (0.4232)	0.194 (0.3743)	0	**0.183** (0.0062)	0.089 (0.4951)
$X_{13}$		0.067 (0.3411)	0.076 (0.6245)	0.076 (0.4823)	0	0.074 (0.2637)
$X_{16}$		0.084 (0.6234)	0.015 (0.4621)	0.085 (0.7231)	**0.072** (0.0214)	0

**Figure A1:**
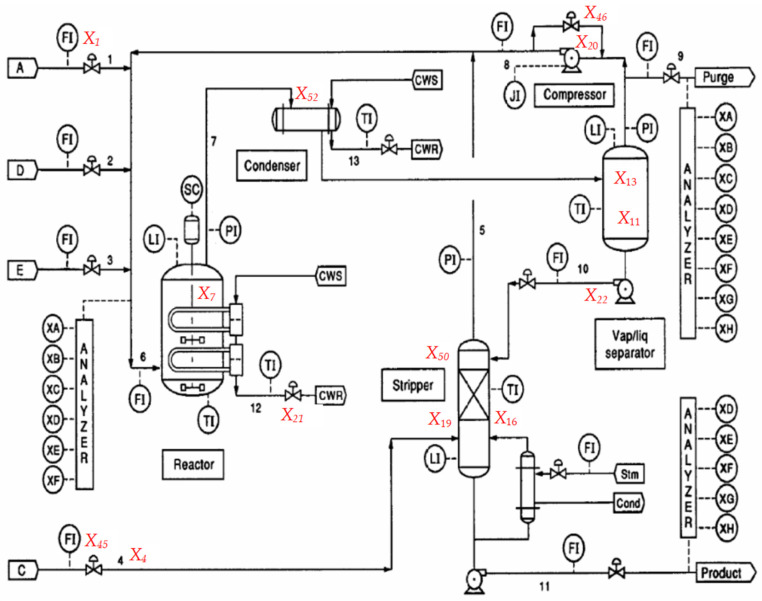
Tennessee Eastman Process.

**Figure A2:**
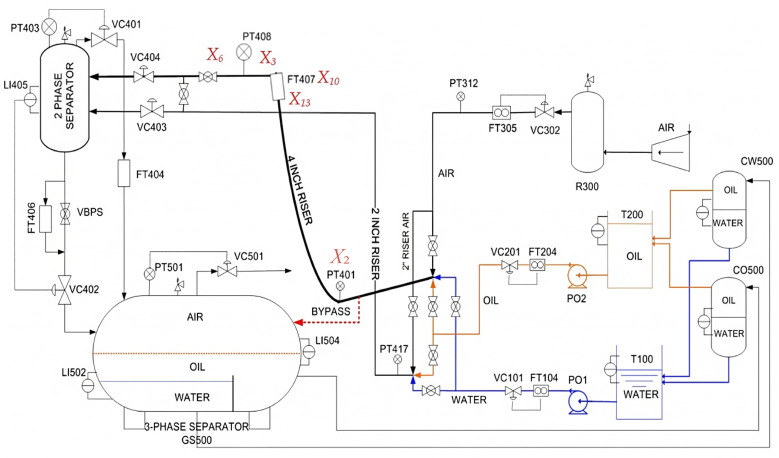
Equipment process flow chart of TPF process.

## Data Availability

The data will be made available on request.

## Appendix A. Tennessee Eastman Process

This section mainly introduces the Tennessee Eastman Process (TEP) used in Section 4.2. The TEP system is a real chemical process simulation model proposed by Downs and Vogel [36] of the Eastman Chemical Company in the United States in 1993. It is widely used in process monitoring and fault diagnosis and is a typical model. The entire TEP has nonlinear and non-stationary characteristics, and the equipment is highly coupled and contains cyclic processes, which makes the fault root cause diagnosis of the TEP extremely challenging. This paper takes the TEP industrial system as an application example for verify the effectiveness of the fault root cause diagnosis method proposed in this paper.

The TEP is a typical chemical production process, including five main pieces of equipment: reactor, condenser, separator, compressor, and stripping tower, as shown in Figure A1.

The production process needs to feed four gaseous raw materials, A, C, D, and E, and inert gas B into the reactor, generating two liquid products, G and H, and a by-product F. The reaction equation of the whole chemical production process is as follows: First, four gaseous raw materials are fed into the reactor to produce a chemical reaction under the action of the catalyst. Next, the products in the reactor and the incompletely reacted feeds, including the inert gas B, are sent to the condenser for cooling. Then, the cooled material is sent to the gas/liquid separator for separation, and the separated steam is passed through a compressor to form a recycling flow, which is put into the reactor again for a reaction to improve the single-pass conversion rate of the reactants. At the same time, in order to prevent the accumulation of by-product F and inert gas B, part of the recycling flow needs to be discharged from the top of the separator. The condensed components separated in the gas/liquid separator (flow 10) are sent to the stripping tower from the bottom of the separator. Stream 4 is used to strip the remaining reaction feed in Stream 10. These remaining feeds are combined with the recycling flow through Stream 5 and enter the reactor again. Finally, products G and H flow out from the bottom of the stripping tower.

In the TEP platform, there are a total of 52 diagnostic variables, including 11 control variables and 41 operating variables. Variables $X_{1}–X_{22}$ are the process measurement variables, which are continuous variables such as temperature, pressure, flow, and liquid level. Variables $X_{23}–X_{41}$ are the component measurement variables, which represent the value of each component and are measured from Stream 6, Stream 9, and Stream 11. Variables $X_{42}–X_{53}$ are the control variables, which are related to the opening of the valve.

## Appendix B. Three-Phase Flow Process

This section mainly introduces the Three-Phase Flow (TPF) dataset. Researchers at Cranfield University set up the TPF device experimental platform and collected relevant experimental data (sampling frequency: 1 HZ) [39,40,41]. This equipment is comparable to a small-scale industrial multiphase flow separation process. Introducing different disturbances into the TPF system simulates possible disturbance scenarios that may occur in the actual system, such as blockage, system operation errors, or unconventional operations. Figure A2 shows the process flow chart of the equipment.

The test area for the TPF process equipment consists of pipes of different apertures and geometries that supply air, water, and oil, as well as mixtures of these fluids, at the required rates. Finally, the fluid mixture is separated in a horizontal three-phase separator (GS500) with a volume of 11 m^3^ on the ground. Air is returned to the atmosphere, and the emulsions of oil and water are separated into their respective condensers (CW500 and CO500, with a capacity of about 1.5 $m^{3}$) before returning to their respective tanks (T200 and T100).

A combination of two compressors supplies air and the compressed air is stored in an 83-inch container (R300) to eliminate the effects of air pressure fluctuations. Water and oil are stored in tanks T100 and T200, respectively, and are independently supplied through multi-stage Grundfos CR90-5 pumps (PO1 and PO2), whose speed is controlled by variable frequency inverters. FT104 measures the water flow, FT204 measures the oil flow, and the water and oil flows are controlled by the pneumatic valves VC101 and VC201, respectively.

After mixing, the fluids can flow through a flow circuit that has a 55-inch-long downward sloping pipe leading to a 10.5-inch-high catenary riser (4 INCH RISER), or through a 40-inch-long horizontal riser connected to a 10.5-inch-long vertical riser (2 INCH RISER). Both flow lines are connected to a two-phase separator, but can be isolated via manual valves at both ends of the flow lines.
